# Succinic acid production on xylose-enriched biorefinery streams by *Actinobacillus succinogenes* in batch fermentation

**DOI:** 10.1186/s13068-016-0425-1

**Published:** 2016-02-02

**Authors:** Davinia Salvachúa, Ali Mohagheghi, Holly Smith, Michael F. A. Bradfield, Willie Nicol, Brenna A. Black, Mary J. Biddy, Nancy Dowe, Gregg T. Beckham

**Affiliations:** National Bioenergy Center, National Renewable Energy Laboratory, Golden, CO 80401 USA; Department of Chemical Engineering, University of Pretoria, Pretoria, South Africa

**Keywords:** Succinic acid, Fermentation, Biochemicals, Biofuels, Pretreatment, Biorefinery, *Actinobacillus succinogenes*

## Abstract

**Background:**

Co-production of chemicals from lignocellulosic biomass alongside fuels holds promise for improving the economic outlook of integrated biorefineries. In current biochemical conversion processes that use thermochemical pretreatment and enzymatic hydrolysis, fractionation of hemicellulose-derived and cellulose-derived sugar streams is possible using hydrothermal or dilute acid pretreatment (DAP), which then offers a route to parallel trains for fuel and chemical production from xylose- and glucose-enriched streams. Succinic acid (SA) is a co-product of particular interest in biorefineries because it could potentially displace petroleum-derived chemicals and polymer precursors for myriad applications. However, SA production from biomass-derived hydrolysates has not yet been fully explored or developed.

**Results:**

Here, we employ *Actinobacillus succinogenes* 130Z to produce succinate in batch fermentations from various substrates including (1) pure sugars to quantify substrate inhibition, (2) from mock hydrolysates similar to those from DAP containing single putative inhibitors, and (3) using the hydrolysate derived from two pilot-scale pretreatments: first, a mild alkaline wash (deacetylation) followed by DAP, and secondly a single DAP step, both with corn stover. These latter streams are both rich in xylose and contain different levels of inhibitors such as acetate, sugar dehydration products (furfural, 5-hydroxymethylfurfural), and lignin-derived products (ferulate, *p*-coumarate). In batch fermentations, we quantify succinate and co-product (acetate and formate) titers as well as succinate yields and productivities. We demonstrate yields of 0.74 g succinate/g sugars and 42.8 g/L succinate from deacetylated DAP hydrolysate, achieving maximum productivities of up to 1.27 g/L-h. Moreover, *A. succinogenes* is shown to detoxify furfural via reduction to furfuryl alcohol, although an initial lag in succinate production is observed when furans are present. Acetate seems to be the main inhibitor for this bacterium present in biomass hydrolysates.

**Conclusion:**

Overall, these results demonstrate that biomass-derived, xylose-enriched hydrolysates result in similar yields and titers but lower productivities compared to clean sugar streams, which can likely be improved via fermentation process developments and metabolic engineering. Overall, this study comprehensively examines the behavior of *A. succinogenes* on xylose-enriched hydrolysates on an industrially relevant, lignocellulosic feedstock, which will pave the way for future work toward eventual SA production in an integrated biorefinery.

**Electronic supplementary material:**

The online version of this article (doi:10.1186/s13068-016-0425-1) contains supplementary material, which is available to authorized users.

## Background

Lignocellulosic biomass has significant potential to serve as a sustainable raw material for the production of renewable fuels and chemicals [1]. The biorefinery concept is an approach that strives to efficiently utilize biomass as a feedstock for integrated biofuels, energy, and chemical production [2, 3]. This approach is analogous to current petroleum refineries wherein myriad products are produced at a single integrated facility. In petroleum refineries, fuel production provides economies of scale which reduces overall costs while the co-production of value-added chemicals substantially enhances the economics and profitability of the process [4]. Similarly, value-added chemicals will be essential for de-risking the economic viability of a lignocellulosic biorefinery, making their co-production crucial in any biorefinery. However, despite numerous reports and reviews cataloging co-products that can be potentially produced from lignocellulosic sugars [5–9], few chemicals are made from lignocellulose-derived sugars today at large scale, although many more chemicals are being manufactured from starch-based sugars. Indeed, many challenges exist for making biochemicals from lignocellulosic sugars, including achieving sufficiently high yields in the conversion step, deploying cost-effective, sustainable separation processes that yield the product at the needed purity and high recovery yields, and competition with petroleum-derived chemicals that often have many more decades of development work behind them.

A candidate value-added co-product for incorporation into a lignocellulosic biorefinery is succinic acid (SA), an aliphatic C4 dicarboxylic acid (butanedioic acid). SA has been identified as a promising biomass-derived, value-added chemical owing to its availability from the biotransformation of biorefinery sugars and its vast potential as a chemical precursor [5, 10, 11, 12, 13, 14]. SA can be catalytically converted to 1,4-butanediol, tetrahydrofuran, and γ-butyrolactone, among other compounds [15]. Traditionally, SA is produced in a petrochemical process via catalytic hydrogenation of maleic anhydride derived from butane [16]. Given that SA (specifically the salt form, succinate) is a primary constituent of the tricarboxylic acid cycle, it can potentially be produced from lignocellulosic sugars at high carbon efficiency. As such, substantial research efforts have been made in the biological production of succinate and its subsequent scale-up [17], the latter which has primarily focused on starch-based sugars to date. However, for bio-based SA not to compete with food resources, it is necessary to utilize cheap lignocellulosic sugars. These feedstocks do not compete with food crops and maintain the advantages of biomass, such as a higher oxygen content (compared to petroleum) [18].

Many studies on bio-based production of SA utilize pure sugars as substrates. In these cases, high yields, titers, and productivities have been achieved with *Anaerobiospirillum succiniciproducens* [19, 20], *A succinogenes* [21–23], engineered strains of *Escherichia coli* [24–27], and *Mannheimia succiniciproducens* [28–30]. Biological production of SA is now also being investigated, albeit to a lesser extent thus far, using lignocellulosic sugars. Representative studies to date include fermentation of the following: corn stalk and sugarcane hydrolysate by engineered *E. coli* [31, 32]; corn stover hydrolysate [33], sugar cane bagasse [34], corn fiber [35], and straw hydrolysate [36] by *A. succinogenes*; cane molasses by modified *A. succinogenes* [37]; and wood hydrolysate by *M. succiniciproducens* [38]. Of these studies, *A. succinogenes* is often a top-performing microbe in terms of succinate titer, rate, and yield. This strain in particular produces succinate naturally in mixed-acid fermentations at relatively high concentrations [39, 40] due partly to its high acid tolerance [41]. Furthermore, it is a non-pathogenic, facultative anaerobe that fixes CO_2_ and consumes a broad range of substrates including C6 (e.g., glucose, galactose) and C5 sugars (e.g., xylose, arabinose) [40]. Since high titer production is important in minimizing downstream separation costs, and as lignocellulosic biomass contains an array of C6 and C5 carbohydrates, *A. succinogenes* is positioned as a promising candidate for industrial succinate production on lignocellulosic feedstocks.

The production of chemicals such as SA in a biorefinery requires close integration and co-development with upstream and downstream unit operations and processes. Biomass deconstruction in particular represents a crucial and often costly step [1]. In conventional biochemical conversion processes, biomass polysaccharides are depolymerized to upgradeable sugars via tandem thermochemical pretreatment followed by enzymatic hydrolysis with cellulase enzymes [1, 42]. Some common pretreatment methods, mainly hydrothermal and dilute acid pretreatment (DAP), are capable of hydrolyzing most of the hemicellulose to produce high yields of monomeric xylose and other hemicellulose-derived sugars [43–49]. Additionally, both aforementioned pretreatment approaches are being deployed currently at the industrial scale in lignocellulosic bioethanol plants. The xylose-enriched stream can be readily fractionated from the remaining cellulose-enriched solids and used in a biorefinery context as a separate process stream for upgrading to either fuels or chemicals. In both acid and hydrothermal pretreatments, many potential microbial inhibitors, such as acetic acid (AA), furfural, hydroxymethylfurfural (HMF), and low molecular weight phenolics are produced [50], and the downstream processes must be able to accommodate these inhibitors or a cost-effective cleanup strategy must be employed.

**Fig. 1:**
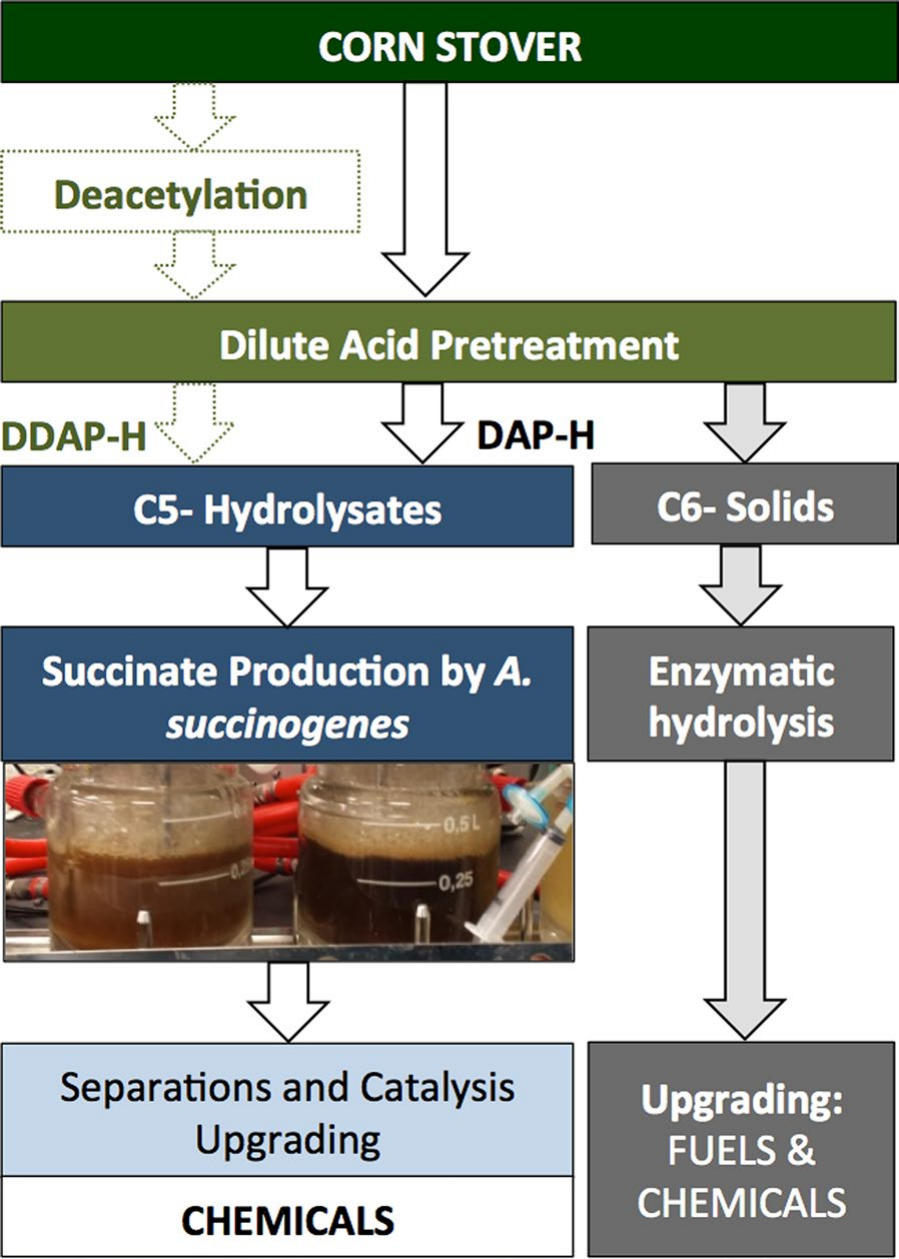
Diagram of the process configurations used to produce succinate by *A. succinogenes* in the current study. Xylose-enriched hydrolysate from corn stover was produced via two separate processes to yield two unique hydrolysates for this work: first, wherein we use a deacetylation step followed by dilute acid pretreatment (DDAP-H) and separately wherein only dilute acid pretreatment (DAP-H) is applied. We note the separations and catalytic upgrading were not performed in the current work. *DAP-H* dilute acid-pretreated hydrolysate; *DDAP-H* deacetylated dilute acid-pretreated hydrolysate

Despite significant and promising work to date on *A. succinogenes* employing biomass hydrolysate as a substrate, work still remains to fully characterize the behavior of the strain and to test succinate production on more process-relevant substrates produced at the pilot-scale. To that end, here we examine the feasibility of producing succinate from pretreated, xylose-enriched corn stover hydrolysate by wild-type *A. succinogenes* 130Z in batch cultures. We examine the behavior of the strain in clean sugar streams and in mixed sugar streams with potential inhibitors added. We then examine succinate production using biomass hydrolysates from two pilot-scale pretreatment processes, namely, a process configuration that first uses a mild alkaline wash, deacetylation, [51, 52], followed by DAP (which we dub “DDAP-H” for deacetylated DAP hydrolysate), and a separate hydrolysate stream that only uses a DAP step with no deacetylation (which we dub “DAP-H” for DAP hydrolysate). In a companion manuscript, we report continuous fermentation of *A. succinogenes* with DDAP-H as well, which exhibits higher productivities [10]. Overall, these studies provide key insights into succinate production via fermentation of xylose-enriched, process-relevant hydrolysates, which in turn represents a step toward the integrated demonstration of large-scale SA production within a biorefinery context (Fig. 1).

## Results

### Pretreatment of corn stover and hydrolysate characterization

Corn stover was pretreated at pilot-scale in two process configurations, illustrated in Fig. 1, namely a DAP step, one without and one with a deacetylation step preceding DAP, as described in detail in the “Methods” section. The primary motivation for using both pretreatments is that deacetylation results in significantly less AA in the hydrolysate [52], which is a potential bacterial inhibitor [50]. Deacetylation likely also removes some of the more labile aromatics present in biomass, such as *p*-coumaric acid and ferulic acid [53, 54]. Thus, we hypothesized that DDAP-H would be less inhibitory to *A. succinogenes.* Corn stover (in the DDAP-H case) was deacetylated at 80 °C for 2 h at a 0.4 % (w/w) NaOH loading. Both the deacetylated material and corn stover underwent DAP at the same conditions, namely with dilute H_2_SO_4_ (8 g H_2_SO_4_/kg of biomass on a dry basis) at 160 °C for 10 min. The composition of both DDAP-H and DAP-H is detailed in Table 1. Xylose was the main sugar in both hydrolysates (~100–115 g/L), followed by arabinose, glucose, and galactose, which sum to 135–156 g/L (total sugar content) in DDAP-H and DAP-H, respectively. As a direct result of the pretreatment, the main difference between both liquors was AA concentration (Table 1). DAP-H contains roughly 7 g/L more AA than DDAP-H. Both hydrolysates also present similar concentrations of two other potential inhibitors namely furfural and HMF, originating from sugar degradation during DAP [50].

**Table 1:** Composition of DDAP-H and DAP-H

Compounds	DDAP-H (g/L)	DAP-H (g/L)
Glucose	13.60	16.90
Xylose	99.00	114.10
Galactose	6.60	8.40
Arabinose	15.60	16.40
Acetic acid	3.80	11.00
Furfural	1.76	1.96
HMF	0.30	0.40

We note that for further fermentations with *A. succinogenes* these hydrolysates are diluted to obtain a total sugar concentration of 80 g/L (~56 % hydrolysate)

### Effect of high xylose and glucose concentrations on succinate production by *A. succinogenes*

**Fig. 2:**
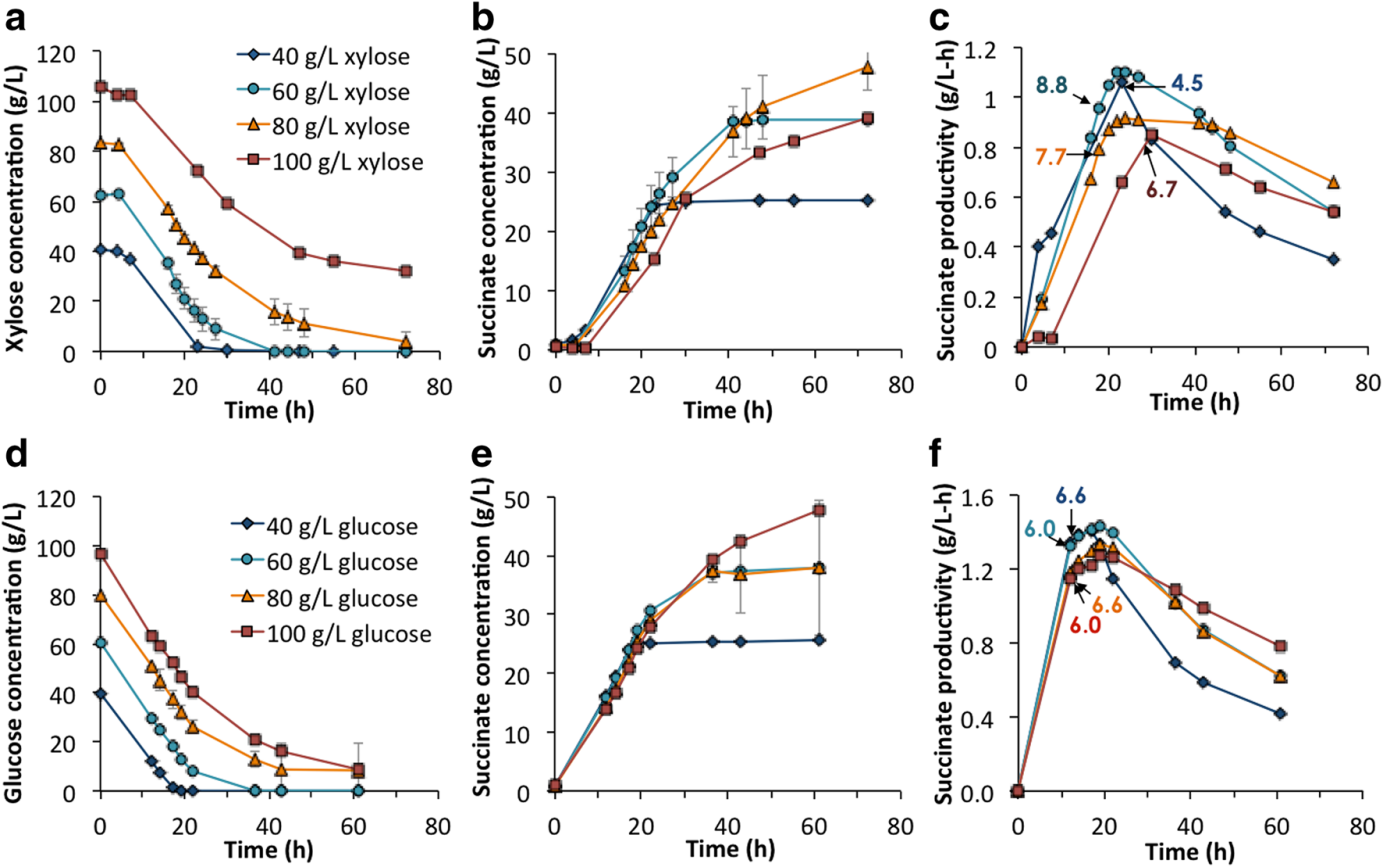
SA production and sugar consumption by *A. succinogenes* in pure xylose and glucose in batch fermentations. Profiles of sugar utilization (**a**, **d**), succinate production (**a**, **e**), and succinate productivity (**c**, **f**) as a function of different initial concentrations of xylose (**a**, **b**, **c**) and glucose (**d**, **e**, **f**). Productivity is calculated as succinate concentration divided by the fermentation time at each point. The *numbers* in **c** and **f** indicate the time point where the maximum cell density (OD_600_) was reached and the specific OD_600_ value for each culture

The inhibition of bacterial growth and succinate production due to high initial glucose concentrations has already been detailed in *A. succinogenes* [41, 55]. However, much less information is found about the effect of high xylose concentrations on this organism. As xylose is the major sugar in acid-pretreated hydrolysates (up to 114 g/L) [43, 44, 45, 51], the first step of this work was to determine the xylose level at which succinate production is inhibited. Thus, we first evaluated xylose consumption and succinate production at different initial xylose concentrations (40, 60, 80–100 g/L) in (Fig. 2a, b). Figure 2a shows that xylose utilization slows when the initial concentration is 100 g/L; indeed, xylose was not fully utilized at this concentration (conversion reaches ~60 % after 72 h of fermentation). At initial concentrations of 40–60 g/L, the sugar was completely utilized at approximately 20–40 h, respectively, which was reflected in a concomitant termination of succinate production (Fig. 2b). At an initial xylose concentration of 80 g/L, the highest succinate titers (48 g/L) were achieved and xylose utilization was 95 % at the end of the fermentation (72 h). Additionally, Fig. 2c shows the succinate productivity as a function of time. In this case, initial xylose concentrations of 80 g/L exhibit lower productivities at the beginning of the incubation time than those found at lower initial xylose concentrations. In fact, maximum productivities were also slightly lower at initial 80 g/L than 60 g/L (0.90 and ~1.1 g/L-h, respectively). In Fig. 2c, the maximum optical density (OD_600_) and the time point in which it was measured is also shown. It is noteworthy how at 80–100 g/L of xylose, the maximum bacterial growth was lower than at 60 g/L (OD_600_ at 40 g/L was probably lower due to an earlier total consumption of the sugars). After reaching the maximum cell biomass, the OD_600_ decreased abruptly in all cases (Additional file 1: Figure S1). *A. succinogenes* is a biofilm-forming microbe [22], and the maximum OD_600_ coincides with the start of the biofilm formation, and thus a decrease in the planktonic cell density in the medium.

Although production of succinate from glucose has been widely studied in *A. succinogenes*, we also performed fermentations in pure glucose to compare the results with the xylose runs on a consistent basis. Figure 2d, e shows the glucose utilization and succinate production at different initial glucose levels (from 40 to 100 g/L). The observed trends differ somewhat to those found with xylose. For instance, glucose utilization and succinate production at an initial 100 g/L glucose concentration did not present the same decrease observed for xylose consumption rates or lag in succinate production as observed at 100 g/L of xylose. Moreover, glucose utilization at the highest concentration was almost complete. The maximum succinate titer (48 g/L) was reached at initial 100 g/L of glucose instead of 80 g/L as with xylose. However, a large deviation in succinate production was observed in the 80 g/L initial glucose concentration case, so we cannot definitively state if succinate production was lower than at 100 g/L. Regarding the productivity (Fig. 2f), differences among the different glucose concentrations were not as evident as with xylose, although productivities at 80–100 g/L were slightly lower than at 40–60 g/L. In terms of cell density, the maximum OD_600_ was also similar among the different treatments, ranging between 6 and 6.6.

### Effect of furfural, HMF, and AA on *A. succinogenes* performance in mock hydrolysates

Hydrolysates from acid pretreatment contain inhibitors that can potentially affect *A. succinogenes* performance [56]. Thus, we studied their effect on the succinate titer and productivity. For this purpose, AA (whose salt form at pH 6.8 is acetate), furfural, and HMF were included in a series of fermentations dubbed “mock” hydrolysates. In the pure xylose fermentations, the experiments conducted at an initial xylose concentration of 80 g/L resulted in the highest succinate titers that do not exhibit substantial substrate inhibition (Fig. 2a). As such, we selected 80 g/L as the final sugar content to prepare the mock hydrolysates. The percentages of each compound in the mock hydrolysates were closely based on the actual composition of the liquors (Table 1) considering the hydrolysate dilution (56 %). Specifically, the different mock media contained: (1) sugars + furfural (1.4 g/L) + HMF (0.17 g/L) + AA (5.8 g/L) (=“Mock DAP-H”), (2) sugars + furfural (1.4 g/L) + HMF (0.17 g/L) + AA (2.3 g/L) (=“Mock DDAP-H”), (3) sugars + AA (5.8 g/L) (=“Mock sugars + AA”), and (4) only sugars (=“Mock sugars”). The sugar mixture contained xylose (58 g/L), glucose (8.7 g/L), arabinose (8.7 g/L), and galactose (4.6 g/L).

**Fig. 3:**
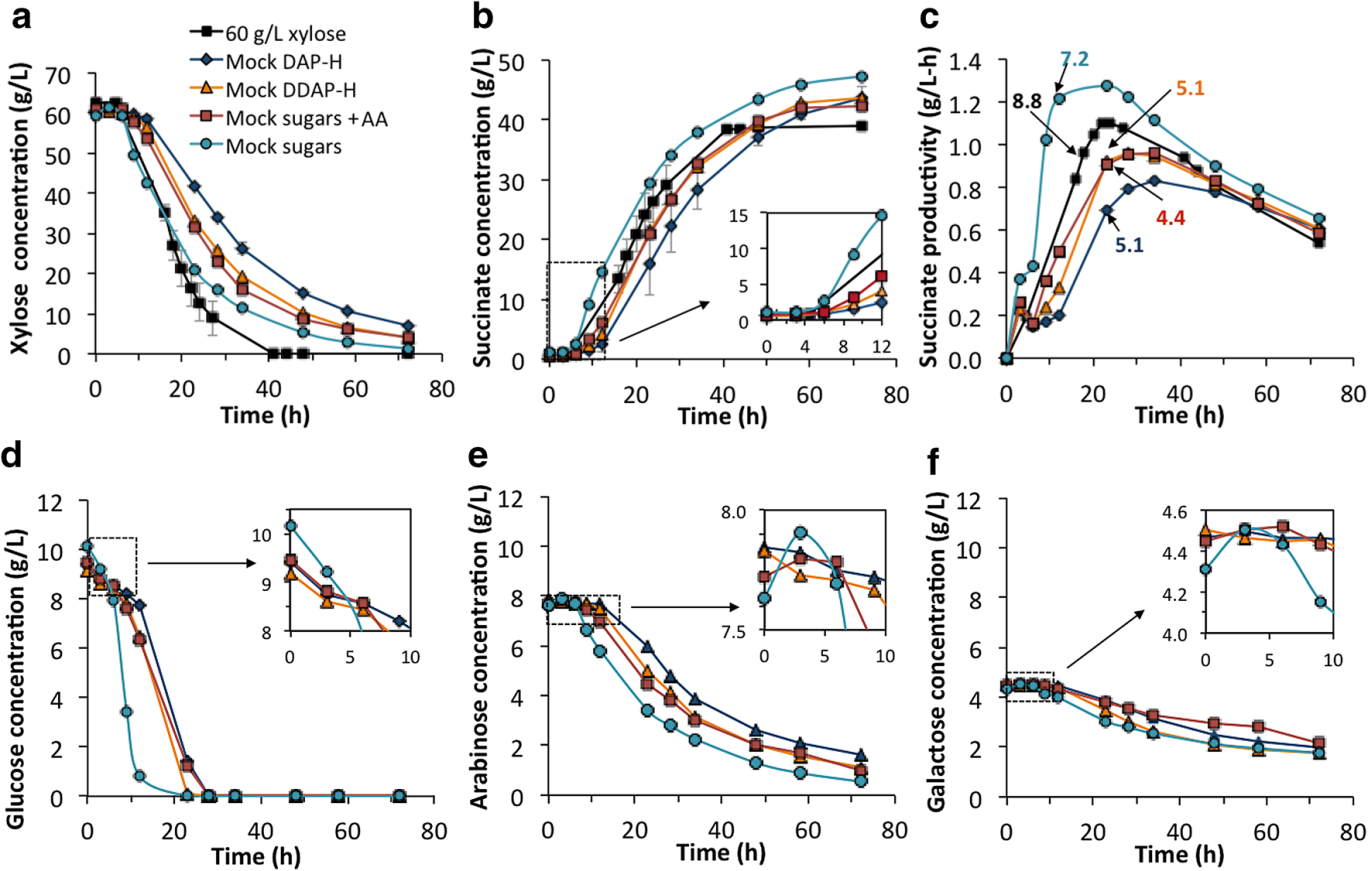
SA production and sugar consumption by *A. succinogenes* in mock media in batch fermentations. Profiles of xylose (**a**) glucose (**d**) arabinose (**e**) and galactose (**f**) consumption, succinate production (**b**) and succinate productivity (**c**) by *A. succinogenes* of different mock hydrolysates. Productivity is calculated as succinate concentration divided by the fermentation time at each point. Sugars and inhibitors in the different media are (1) mock DAP-H = sugars (80 g/L) + furfural (1.7 g/L) + HMF (0.17 g/L) + AA (5.8 g/L), (2) mock DDAP-H = sugars (80 g/L) + furfural (1.7 g/L) + HMF (0.17 g/L) + AA (2.3 g/L), (3) mock sugars + AA = sugars (80 g/L) + AA (5.8 g/L), and (4) mock sugars = only sugars (80 g/L). *Insets* in the *graphs* **b**, **d**, **e**, and **f** present the profiles corresponding to the first few hours of fermentation. The *numbers* in **c** indicate the time point where the maximum cell density (OD_600_) was reached and the specific OD_600_ value for each culture

Figure 3a, b show the profile for xylose consumption and succinate production in the different mock hydrolysates, respectively. As seen from these figures, the consumption of xylose in “mock sugars” was slower than in the pure xylose fermentation (at the same initial xylose concentration of ~60 g/L) in previous experiments (Fig. 2a). However, succinate production was enhanced in the former case, likely because there were more sugars present than xylose, including glucose. Regarding productivity, the “mock sugars” unsurprisingly exhibited the highest maximum productivity in the current set of fermentations (> 1.2 g/L-h) (Fig. 3c). In fact, the maximum productivity was improved relatively to pure xylose fermentations (Fig. 2c), likely as glucose was utilized at higher rates by *A. succinogenes*. Comparing “mock sugars” with “mock sugars + AA,” an obvious initial lag for xylose consumption and succinate production was observed in the latter case as well as a significant decrease in the productivity. Interestingly, the profile obtained in “mock sugars + AA” was also very similar to the one found for “mock DDAP-H.” Acetate concentration in the former case was higher than in “mock DDAP-H” (since it was mimicking AA concentration in DAP-H). These data demonstrate that higher concentrations of AA (5.8 g/L) were as inhibitory as lower AA concentrations (2.3 g/L) along with furfural and HMF. Lastly, and as expected considering the previous results, xylose utilization and succinate production in “mock DAP-H” was further delayed compared to the other treatments and thus, the productivity was lower. However, titers at the end of the incubation time were similar in all cases reaching values between 47 g/L in “mock sugars” and 42 g/L in “mock sugars + AA.” Regarding the maximum cell density, a substantial difference among “mock sugars” (OD_600_ = 7.2) and the rest of the mock hydrolysates (OD_600_ between 4.4 and 5.1) was detected. These data show that bacterial growth was not highly correlated with final succinate titers in batch fermentation suggesting that there were other mechanisms of inhibition that hindered succinate production titers (e.g., high concentration of acids in the fermentation broth).

In the experiment shown in Fig. 3, the utilization of glucose, arabinose, and galactose was also tracked (Fig. 3d, e). In terms of utilization, trends among the different mock hydrolysates were similar to those reported for xylose in the same experiment, demonstrating again the fastest sugar utilization rates in “mock sugars.” Comparing the initial utilization rates, we can also suggest the sugar preferences in *A. succinogenes*. It was quite clear among the substrates tested that this bacterium preferentially metabolizes glucose. The glucose consumption rate during the first 3 h of fermentation was 0.25 g/L-h (in a period of time where the other 3 sugars had not yet started to be metabolized). Subsequently, during the first 9 h, although all sugars were utilized simultaneously, the consumption rates for xylose, arabinose, and galactose were 1.08, 0.11, and 0.02 g/L-h, respectively. We also noted how galactose utilization was quite slow and not complete by the end of the fermentation, with a final conversion of about 60 % after 72 h.

### Production of succinate in DAP-H and DDAP-H

**Fig. 4:**
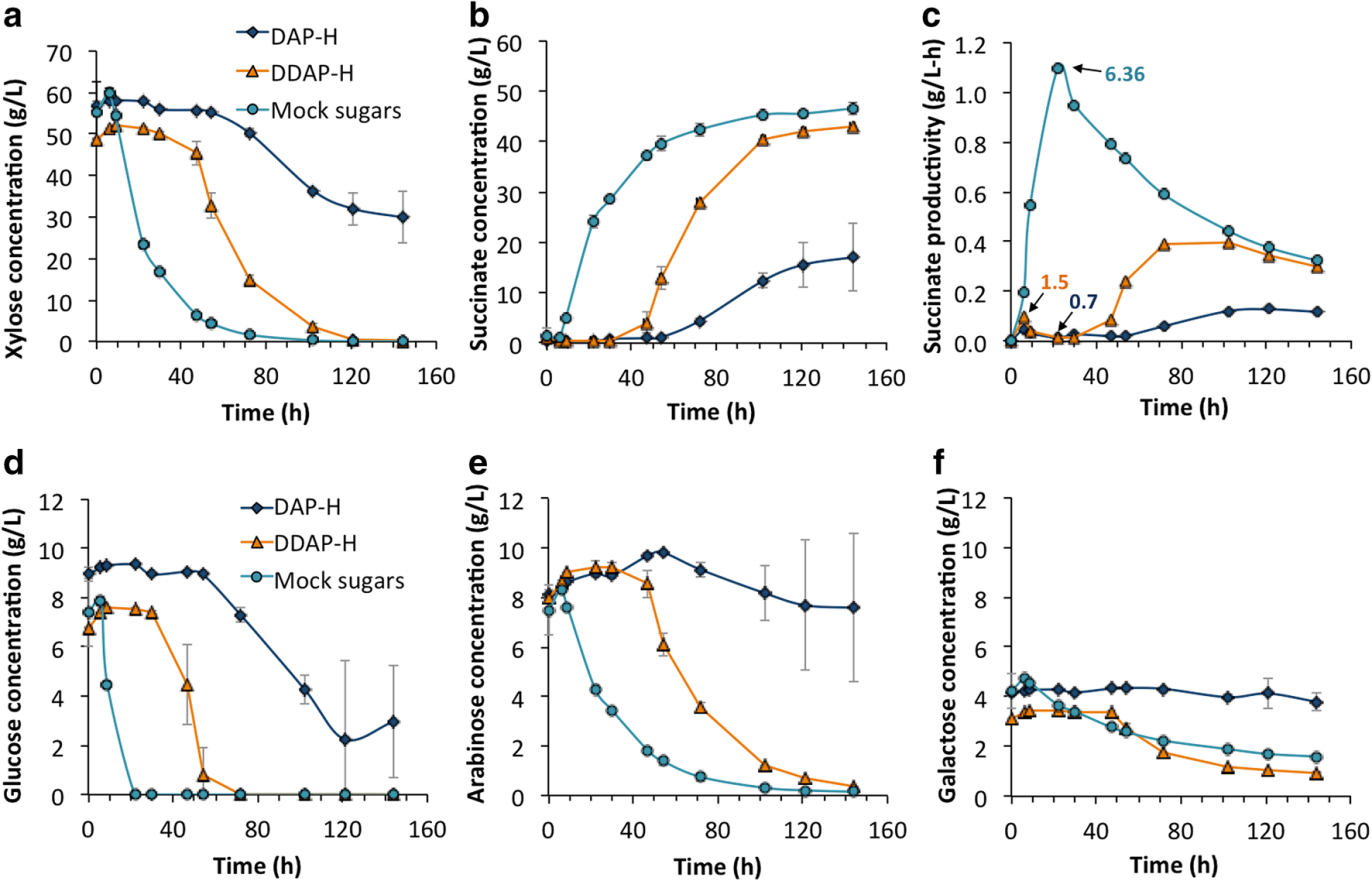
SA production and sugar utilization by *A. succinogenes* in DAP-H and DDAP-H in batch fermentations. Profiles of xylose (**a**), glucose (**d**), arabinose (**e**), and galactose (**f**) consumption, succinate production (**b**), and succinate productivity (**c**) by *A. succinogenes* in batch fermentations in mock sugars and DAP-H and DDAP-H. Productivity is calculated as succinate concentration divided by the fermentation time at each point. The *numbers* in **c** indicate the time point where the maximum cell density (OD_600_) was reached and the specific OD_600_ value for each culture

As *A. succinogenes* is able to grow and produce succinate in mock hydrolysates in the presence of acetate, furfural, and HMF, we next evaluated *A. succinogenes* performance in DAP-H and DDAP-H. These hydrolysates were also diluted to an initial sugar concentration of approximately 80 g/L (56 % hydrolysate). Figure 4 shows the profiles of sugar consumption, succinate production, and succinate productivity. For this set of fermentations, we used the “mock sugars” as a control. As observed, there was a long initial lag phase in both hydrolysates for sugar utilization and consequently in succinate production. Succinate production (Fig. 4b) did not commence until glucose utilization began at 30–54 h in DDAP-H and DAP-H, respectively (Fig. 4d). From these fermentation time points onward, succinate production (Fig. 4b) and productivity (Fig. 4c) increased significantly, together with a meaningful conversion of the other sugars (after 47–72 h in DDAP-H and DAP-H, respectively) (Fig. 4a, e, f). Here, we similarly observe that despite the initial lag in DDAP-H, the final succinate titer (43 g/L) was similar to that obtained in the “mock sugars” hydrolysate (47 g/L). Succinate titers in DAP-H were much lower, likely due to the slow, incomplete conversion of the sugars. From these experiments, we can also observe important differences between “mock DDAP-H” and DDAP-H. For instance, succinate production started at ~10 h in the former (Fig. 3b) and at ~47 h in the latter (Fig. 4b). Moreover, the productivity decreased due to the initial lag, from ~1 (Fig. 3c) to ~0.4 g/L-h (Fig. 4c). Conversely, differences between “mock DAP-H” and DAP-H are much more drastic than in the previous case. These results suggest that there must be other potent inhibitors in the hydrolysate that were not considered at the time of preparing the mock hydrolysates (e.g., phenolic compounds or Xylo-oligosaccharides) [57]. Regarding cell density, a maximum OD_600_ of 1.5 at 9 h for DDAP-H and OD_600_ of 0.7 at 22 h for DAP-H were measured, although these results are not accurate due to the early formation of bacterial aggregates in these fermentations, likely due to the hydrolysate toxicity.

### Bacterial metabolism of inhibitory compounds and co-product generation during *A. succinogenes* fermentation in DAP-H and DDAP-H

**Fig. 5:**
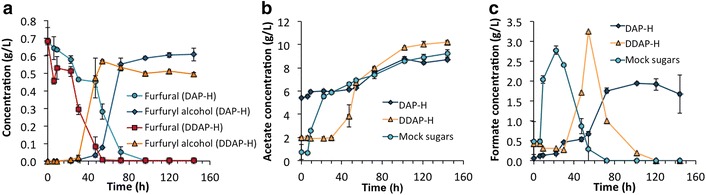
Inhibitors and co-products metabolism during *A. succinogenes* fermentation. Profiles of (**a**) furfural conversion to furfuryl alcohol in DAP-H and DDAP-H and (**b**) acetate production and (**c**) formate production and metabolism in mock sugars, DAP-H, and DDAP-H by *A. succinogenes* in batch fermentation

Considering the previous results, the presence of inhibitors in the hydrolysate affects one of the most important parameters for succinate commercialization, namely productivity. Thus, a detailed analysis of the metabolism of furfural and HMF (Fig. 5a, b) and acetate was performed. Furfural and HMF both disappear during the fermentation. It has been previously reported that these compounds can be converted to alcohols (furfuryl alcohol and HMF-alcohol, respectively) in anaerobic conditions by other organisms [58]. With this precedent, we searched for both alcohols in the fermentation broths. HMF-alcohol was not found, probably because initial HMF concentrations were already quite low. In contrast, in the case of furfural, conversion to furfuryl alcohol was detected (Fig. 5a). Furfural in DAP-H was metabolized later than in DDAP-H. (Additional file 1: Figure S2) also shows the total conversion of furfural and HMF in “mock DAP-H” and “mock DDAP-H,” which occurred faster than in the actual hydrolysates.

Regarding acetate, it is noteworthy that *A. succinogenes* does not utilize acetate from the hydrolysate as other bacteria do [59, 60], but rather it produces high levels of this compound as result of its metabolism. In the current study, acetate levels at the end of all the fermentations were similar (between 8 and 10 g/L) and were independent of the initial concentration of acetate at the start of the fermentation (Fig. 5b). Similar trends were observed in fermentations in mock media in the presence of different concentrations of acetate (Additional file 1: Figure S3). In the case of formate (another product of *A. succinogenes* metabolism), a different trend was observed. Although this acid was a product of the bacterial metabolism (reaching values up to 3.5 g/L), it can be consecutively metabolized probably due to the microbe’s ability to synthesize the formate dehydrogenase enzyme (Fig. 5b). Formate production as well as its “disappearance” was slower in DDAP-H and DAP-H than in mock sugars; indeed, formate was not completely metabolized in DAP-H. (Additional file 1: Figure S4) shows the production of formate in the other fermentation experiments in pure sugars and mock media, which exhibits similar trends and maximum concentrations up to 5.5 g/L.

As introduced in the previous section and in addition to the previously detailed inhibitors, there are clearly other compounds present in the biomass-derived hydrolysates that influence the succinate productivity and bacterial growth. Phenolic compounds, derived from lignin and hemicellulose-lignin linkages, can also be present in the hydrolysates and may also act as inhibitors [50, 57]. Thus, the concentrations of aromatic compounds were measured (Table 2). Their concentrations are in the order of mg/L, with the main aromatic compounds being ferulate (12.6 mg/L) and *p*-coumarate (3.5 mg/L) in DAP-H. These concentrations were slightly lower in DDAP-H (6.9 and 2.0 mg/L, respectively).

**Table 2:** Phenolic compounds in DAP-H and DDAP-H

Phenolic compounds	DDAP-H (mg/L)	DAP-H (mg/L)
*p*-Coumarate	2.0 ± 0.1	3.5 ± 0.2
Ferulate	6.9 ± 1.1	12.6 ± 1.4
4-Hydroxybenzaldehyde	0.7 ± 0.1	0.9 ± 0.0
Caffeate	0.0 ± 0.0	0.0 ± 0.0

### SA yields and maximum productivities from the fermentations in the different media

**Fig. 6:**
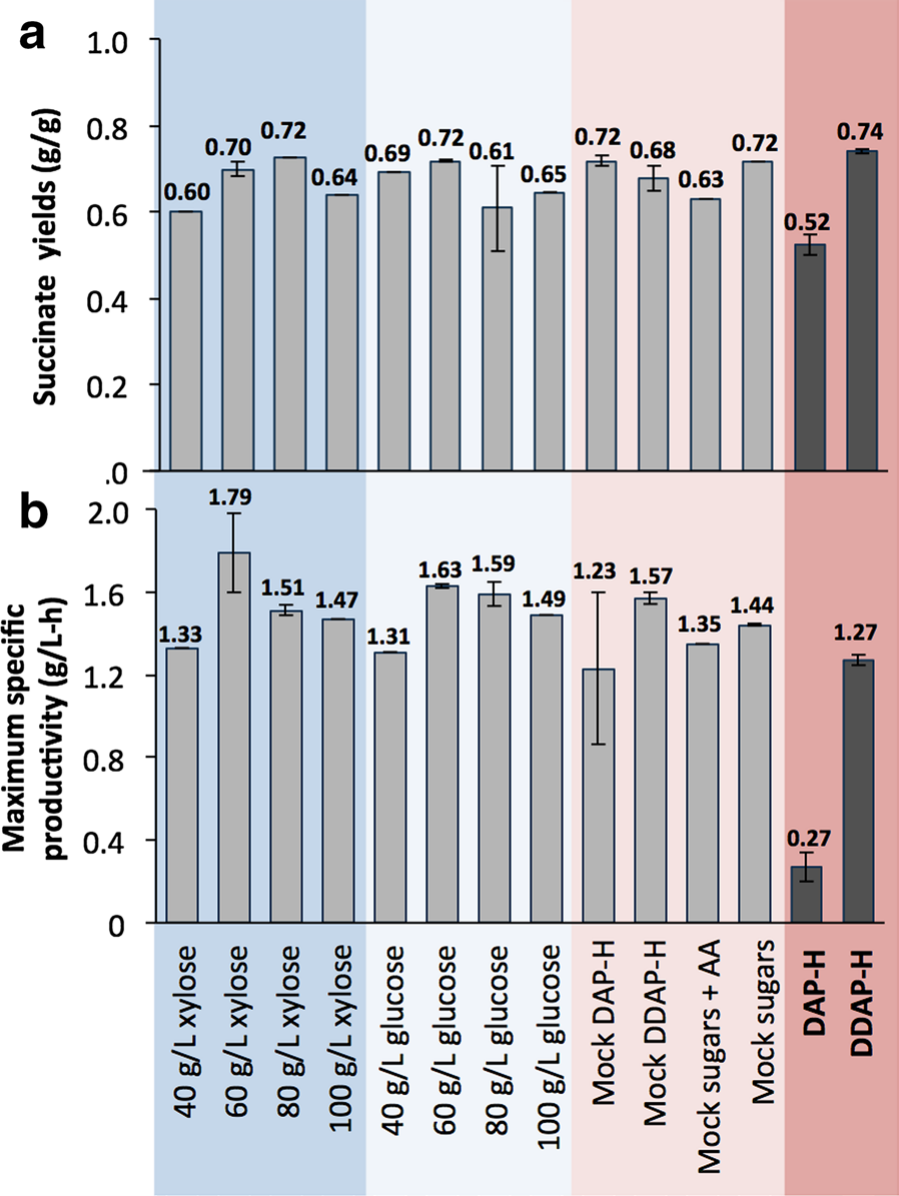
Succinate yields (g succinate/g sugars) and maximum productivities (g/L-h) from all of the batch fermentations conducted in this work. Maximum specific productivities are calculated from the time interval that exhibits the highest slope in the succinate titer profiles. Those time intervals were specifically (1) for all glucose runs and concentrations from 12 to 19 h, (2) for 40 g/L xylose from 7 to 23 h, for 60–80 g/L xylose from 16 to 22 h, and for 100 g/L from 23 to 30 h, (3) for “mock DAP-H,” “mock DDAP-H,” and “mock sugars + AA” from 12 to 23 h and for “mock sugars” from 9 to 23 h, and lastly (4) for DAP-H from 72 to 102 h and for DDAP-H from 47 to 54 h. Yields were based on mass calculations that incorporate Na_2_CO_3_ dilution and sampling volumes removed from the fermentor. Standard deviations for yields were less than 5 % (excluding the treatment with glucose 80 g/L where it was 16 %). Standard deviations for productivities were less than 10 % (excluding in mock DAP-H which was 29 %)

As previously mentioned, achieving high yields, titers, and productivities are essential parameters for product commercialization. Thus far, both succinate titers and productivities have been reported and calculated as detailed in “Methods” section. In Fig. 6, we present the overall yields from all the fermentation runs and the maximum specific productivities in each case. We note that the overall maximum theoretical yield of succinate from glucose is 1.12 g/g [61]. Succinate yields ranged between 0.60 and 0.74 g/g (Fig. 6a), excluding the lowest yield obtained in DAP-H (0.52 g/g) due to incomplete sugar utilization. The maximum yield obtained in this work was in DDAP-H (0.74 g/g). Yield decreases, compared to the theoretical values, are mainly due to the generation of other co-products by the bacterium such as acetate and formate and also due to bacterial biomass formation.

Maximum specific productivities are calculated from the intervals of time where slopes in succinate titer reach a maximum (Fig. 6b). The lowest values are found in both “mock DAP-H” (1.23 g/L-h) and DAP-H (0.27 g/L-h) and the highest in pure xylose at an initial xylose concentration of 60 g/L (1.79 g/L-h). In contrast to the yield results in Fig. 6a, the maximum productivity in DDAP-H (1.27 g/L-h) was lower than in most of the treatments. This result suggests that apart from the initial lag for sugar utilization (Fig. 4a), reflected as productivity decreases (Fig. 5c), the efficiency of *A. succinogenes* producing succinate in DDAP-H was also somewhat reduced compared to pure sugars or the equivalent mock hydrolysate with acetate, furfural, and HMF added.

## Discussion and conclusions

Given the high cost of lignocellulosic feedstocks, the modern lignocellulosic biorefinery will likely need to produce value-added co-products alongside fuels, either from carbohydrate streams or potentially from the lignin fraction of biomass [47, 62]. Figure 1 illustrates a potential route to produce a xylose-enriched stream in the context of dilute acid or hydrothermal pretreatment processes that could be separated from the cellulose-enriched solids [63] and upgraded to, for example, SA as a co-product with higher value than ethanol or a hydrocarbon fuel. To investigate the feasibility of this overall approach, integrated studies are required that employ biomass hydrolysates from different pretreatment conditions. Here we demonstrate that deacetylation prior to DAP has a substantial, positive impact on the ability to produce succinate, similar to the positive impacts on ethanol production in *Zymomonas mobilis* [52].

The main feature of the biomass-derived hydrolysates used in the current study is their high xylose content. As previously described, inhibition due to high glucose concentration has been reported for *A. succinogenes* [41, 55] but not systematically for xylose. Moreover, the studies that have reported the production of succinate from xylose-enriched hydrolysates do not analyze the inhibition levels, or use lower xylose concentrations [34, 64, 65]. Consequently, we first conducted fermentations at different xylose concentrations to determine the limit of sugar inhibition. In parallel, we performed the same study with glucose for comparison purposes. Results show that inhibition by high xylose concentrations was more evident than for glucose, noted by a longer initial lag period on xylose during bacterial growth (Additional file 1: Figure S1A) and succinate production (Fig. 2b). Despite this, succinate yields and titers were higher at initial xylose concentrations of 80 g/L than at 40–60 g/L, and similar to those reached with glucose (Fig. 6). In view of these results, considering the best succinate titers attained in xylose runs and also the substantial decrease of succinate productivity at 100 g/L (Fig. 2c), we selected 80 g/L as the final sugar concentration for further experiments in mock hydrolysates. Xylose uptake in mock hydrolysates (Figs. 3a, 4a) was slower than in pure xylose. For instance, in “mock sugars,” this could be due to the presence of other sugars for which *A. succinogenes* has utilization preference over xylose (e.g., glucose). In the case of the biomass-derived hydrolysates, the presence of inhibitors is likely generating the significant initial lag in xylose utilization. Concerning the utilization of different sugars by *A. succinogenes*, we also described how this bacterium uptakes glucose first and then simultaneously xylose, arabinose, and galactose at different rates. It is known that *A. succinogenes* is able to consume all these sugars [40, 66] but, in addition, our results clearly demonstrate the utilization rates and patterns using the sugar concentrations found in actual biomass-derived hydrolysates (Figs. 3d–f, 4d–f). Increasing the metabolic rates of these sugars is an attractive target for further metabolic engineering since arabinose and galactose constitute 22–25 g/L in DAP hydrolysates and the efficient consumption of these sugars could considerably increase SA production and impact the economic viability of SA in a biorefinery.

Bacterial biomass was also a fundamental parameter studied in fermentation processes since it can directly influence succinate production levels. *A. succinogenes* is a biofilm-forming organism [22], which makes it difficult to track bacterial growth with OD_600_. Moreover, when hydrolysates were used instead of pure sugars, bacterial aggregates were observed much earlier during the fermentation. Regardless, we tracked the OD_600_ of the “free cells” in the broth to determine when the biofilm forms. Biofilm formation, denoted by a drop in OD_600_, occurs earlier in pure glucose and “mock-sugar” fermentations (~12 h) than in pure xylose and other mock hydrolysates (~18–23 h) (Additional file 1: Figure S1). In terms of maximum OD_600_, xylose gave the highest values (OD_600_ = 8.8), followed by mock sugars (OD_600_ = 7.2), glucose (OD_600_ = 6.6), and the other mock hydrolysates. Moreover, OD_600_ also decreased in parallel to the increase of initial xylose concentration from an OD_600_ of 8.8 to 6.7 at 60–100 g/L concentrations, respectively. Liu et al. [37] also reported how bacterial biomass is lower when sugar concentration increases using cane molasses, likely due to inhibition [67] at high sugar concentrations (or acid content, as explained below). From these results, we also note that succinate productivity decreases after biofilm formation (Figs. 2c,f, 3c), although succinate production does not cease (Figs. 2b,f,  3b). Similar effects have been reported by Corona-Gonzalez et al. [55] and Van Heerden et al. [61], namely, that despite cell growth termination, succinate production continues in the presence of available glucose, suggesting that non-growing cells are metabolically active.

Inhibition due to high concentration of acids produced during fermentation (or added from pretreatment) was another parameter considered in the current study. Acids produced by *A. succinogenes* mainly consist of succinic acid, with acetic and formic acids as the primary side products. The maximum acid concentrations at which *A. succinogenes* can survive have been reported as 104, 46, and 16 g/L for SA, AA, and formic acid, respectively [41]. In the current work, these acid levels were not reached, but inhibition (mainly translated as decrease in succinate productivity or maximum bacterial growth) could be observed at much lower concentrations. Corona et al. [55, 68] described that when total acids reach 20 g/L, glucose utilization rates decrease and growth ceases. We observed similar results in xylose, glucose, and mock hydrolysate fermentations, where at approximately 25–30 g/L of total acids (~15–20 g/L succinate, ~6 g/L acetate, and ~4 g/L of formate), biofilm formation initiates. With this precedent and also considering the incomplete utilization of xylose when the initial concentration was 100 g/L, these results point to a more pronounced inhibition of growth by products than the sugar concentration. As previously described, the formation of aggregates in DAP-H and DDAP-H was earlier than the production of 25–30 g/L of total acids. This suggests that biofilm formation was not only due to high acid levels, but also likely due to other compounds present in the hydrolysate inducing a stress response, which results in biofilm formation.

The ratios of succinate/acetate and succinate/formate are important indicators of the bacterial metabolism, and as such, were also tracked during the fermentations. The succinate/acetate ratio increased over time in all cases and also when xylose and glucose concentrations increased (succinate/acetate = 2.3–3 at initial 40 g/L of sugars and succinate/acetate = 6 at 100 g/L both at the end point). These increases have already been reported and are directly linked with formate depletion [37, 69]. The ratios for mock hydrolysates at the end point were 2.9, 3.4, 4.1, and 4.0 for “mock DAP-H,” “mock DDAP-H,” “mock sugars + AA,” and “mock sugars,” respectively. For DAP-H and DDAP-H, succinate/acetate ratios were 2.0 and 4.2, respectively. It is interesting to see how the ratio was the same for mock sugars in the presence and the absence of acetate or even compared to DDAP-H. In theory, this ratio should be 3.94 g/g when all formate is depleted (Fig. 5c) [22], which agrees with these results, being around 4 in “mock sugars” and DDAP-H cultures and meaningfully lower in DAP-H. Moreover, considering all the data, it seems that the final concentration of acetate was not highly dependent on the initial concentration of acetate in the hydrolysate (Additional file 1: Figure S3, Fig. 5b). In the case of the succinate/formate ratio, values also increased quite considerably over time. Moreover, formate was depleted in some cases during the fermentation (Fig. 5c). Interestingly, in the mock hydrolysate experiments, formate seemed to be metabolized faster and totally consumed in treatments in the presence of higher acetate concentration (Additional file 1: Figure S4). In the production/metabolism profile of formate, we can see that it reaches a maximum (between 3 and 5.5 g/L) in all cases, which mostly coincides with the maximum OD_600_ in each treatment. After these maximum peaks, formate decreases abruptly since the bacterium may be metabolizing it to CO_2_ and H_2_O producing NADH [69]; in parallel, the biofilm begins to form. The generation of that NADH can continue supporting succinate production during the fermentation. In general, the productivity of all the acids decreased after the growth termination, supporting the idea of the growth and maintenance modes dubbed by Brink et al. [69].

Hydrolysates from biomass can contain other compounds that act as inhibitors for *A. succinogenes* growth or succinate production such as furfural, HMF, and acetate. In the current study, the effect of furfural and HMF was also studied by using mock hydrolysates separately to DAP-H and DDAP-H. Although they contain the same furfural and HMF concentration, “mock DAP-H” was more inhibitory for the bacterium than “mock DDAP-H,” due to the higher concentration of acetate. Interestingly, however, “mock sugar + AA” (which contains high acetate concentration) presented very similar profiles for sugar utilization, succinate production, and succinate productivities to “mock DDAP-H” (Fig. 3). This fact shows that inhibition levels are a combined effect between furfural, HMF, and acetate. In the current work, apart from characterizing the initial concentrations of furfural and HMF in the hydrolysates, as other studies have [34, 65], their concentrations were also tracked over time. Interestingly, furfural was consumed after 54 and 96 h in DAP-H and DDAP-H, respectively (Fig. 5a) as well as HMF (data not shown). The conversion of these inhibitors to furfuryl alcohol and HMF-alcohol in anaerobic conditions has been previously reported for other organisms [58, 70, 71]. Furfuryl alcohol was detected and its production parallels the decrease of furfural (Fig. 5a). As previously mentioned, high acetate concentrations in DAP-H slows the metabolism of inhibitors like furfural. It is noteworthy that xylose and other sugars were not consumed at high rates until furfural is completely reduced (Figs. 4a, 5a).

Lastly, we compare our results (titers, yields, and productivity) with those reported in the literature utilizing *A. succinogenes* to produce SA from biomass. In the current study, the titer reached in DDAP-H was 43 g/L, which was higher than the titer obtained in DAP-H (Fig. 4b), slightly lower than the maximum obtained in pure sugars (48 g/L), and equal to that achieved in “mock DDAP-H.” Regarding yields, the maximum was reached in DDAP-H at 0.74 g succinate/g sugars (Fig. 6), which was similar to the maximum obtained in pure sugars (~0.72 g/g). The maximum specific productivity for DDAP-H and DAP-H were 1.27 and 0.27 g/L-h, respectively (Fig. 6), although the relative productivities were much lower than in the rest of the treatments due to the initial lag (Fig. 4c). In view of these data, the productivity was the main parameter negatively affected when using hydrolysate. The performance of *A. succinogenes* was also tested in different straw hydrolysates, with the best results reported in corn straw, reaching titers up to 45.4 g/L, a yield of 0.9 g/g, and productivity of 0.95 g/L-h in batch fermentation and an initial sugar content of 58 g/L [36]. The same experiments were performed in fed-batch fermentation, finding enhancements in titers up to 53 g/L and productivities 1.21 g/L. However, these hydrolysates contained significantly more glucose than xylose and as we have seen, productivities can be improved in the presence of glucose. Similar studies to our work (in terms of using xylose-enriched hydrolysates) were performed with corncob [65] and sugarcane bagasse [34, 64]. From corncob hydrolysate with an initial concentration of 34 g/L of xylose, the yields obtained were 0.58 g succinate/g sugars with titers of 23.6 g/L [65]. From sugarcane bagasse hemicellulose hydrolysate, the titer was 22.5 g/L from an initial sugar concentration of 52 g/L with a corresponding yield of 0.62 g/g and productivity of 1.02 g/L-h [64]. Lastly, in sugarcane bagasse after ultrasonic pretreatment, the titer reached was 23.7 g/L with a 79 % (0.88 g/g) yield and productivity of 0.99 g/L-h from an initial concentration of 50 g/L sugars [34]. Considering these findings, our current results exhibit one of the highest succinate titers and maximum specific productivities from lignocellulosic feedstocks obtained to date in batch fermentation processes.

For commercial production of SA from lignocellulosic hydrolysate, even higher titers, productivities, and yields must be reached. To enhance the feasibility of producing SA from these hydrolysates, our next investigations will focus on increasing yields and titers (through metabolic engineering) and improving titers and productivities (mainly by changing fermentation strategies). Continuous fermentation utilizing *A. succinogenes* is reported in a companion manuscript, which results in significantly higher succinate productivities from DDAP-H, reaching values up to 1.77 g/L-h [10]. Some reported values from continuous fermentation range from 6.35 g/L-h [61], 7 g/L-h [72], and 10.8 g/L-h [21], all using glucose as the main carbon source. Repeated-batch fermentation will also be considered, since the highest reported succinate titers (98.7 g/L) have been obtained using that mode [23] although productivity was lower than in continuous fermentations (2.77 g/L-h). Given very promising initial results reported here, continued fermentation optimization and metabolic engineering will both be pursued to develop an optimal SA production process from xylose-enriched lignocellulosic hydrolysates using *A. succinogenes* as a microbial host.

## Methods

### Pretreatment of corn stover and preparation of the hydrolysate

Corn stover was provided by Idaho National Labs (INL Lot #5). Corn stover was knife-milled, sieved through ¾˝ screen, and pretreated with diluted H_2_SO_4_ (concentration 8 g sulfuric acid per kilogram of biomass) at 160 °C for 10 min with the residence time based on the assumption of plug flow in the reactor. Dilute acid pretreatment was performed in 1 ton/day continuous horizontal reactor in both deacetylated (process explained below) and non-deacetylated corn stover.

Deacetylation of corn stover was performed at 8 % (w/w) total solids (TS) concentration with 1500 kg total mass at 80 °C, 2 h, and 0.4 % (w/w) NaOH in the NREL Dynamic Impregnator (DI) vessel. The DI was mixed at 15 rpm during deacetylation. After deacetylation, the spent caustic liquor was drained from the vessel, leaving the remaining solids at 12 % TS. The remaining solids were rinsed with 950 kg of water, which was drained from the vessel and discarded. Solids were then subjected to the dilute acid pretreatment (detailed above).

Pretreated deacetylated and non-deacetylated solids were pressed to obtain the hydrolysate. The pH of the hydrolysate was around 1.9 and was neutralized by NaOH (10 N) as needed for the fermentation assays. Then, the hydrolysate was filter-sterilized. Pretreated corn stover was stored at 4 °C prior to further processing.

### Microorganism and growth conditions

*Actinobacillus succinogenes* 130Z (ATCC 55618) was used for this study. Cells were anaerobically grown in sterile capped bottles (100 mL) containing 50 mL of Tryptic soy broth (Fluka Analytical, India) and incubated overnight at 37 °C and 200 rpm. Cells were harvested by centrifugation (Sorvall), then resuspended in 5 mL Tryptic soy broth and 5 mL glycerol, aliquoted in cryovials, and stored at −70 °C. Prior to the inoculum preparation, bacteria were revived from the glycerol stock at the same conditions detailed above. Bacterial growth was followed by optical density measurements at 600 nm (OD_600_).

### Inoculum preparation

To prepare a large volume of bacterial inoculum, a revived bacterial culture was transferred into a fermentor with tryptic soy broth and 4 % glucose (as an exception, in the pure xylose fermentation runs 4 % xylose was added) at an initial OD_600_ of 0.05. This fermentation was performed overnight at the same conditions as those detailed below (fermentation conditions).

### Fermentation media and experimental design

The media used for fermentations contained (per liter): nutrient source (6 g yeast extract, 10 g corn steep liquor (Sigma-Aldrich, USA) prepared as described below), phosphates solution (0.3 g Na_2_HPO_4_, 1.4 g NaH_2_PO_4_, 1.5 g K_2_HPO_4_), and salt solution (1.4 g sodium acetate, 1 g NaCl, 0.2 g MgCl_2_·6H_2_O, and 0.2 g CaCl_2_·2H_2_O). All solutions were prepared as 10X stock, sterilized by filtration, and then mixed when setting up the fermenters. Phosphate solution was added immediately before the inoculation to avoid precipitation. Corn steep liquor was prepared at a concentration of 200 g/L (20X) and then boiled at 105 °C for 15 min [22]. After cooling, solids were separated and the supernatant was autoclaved and used as nutrient source. As a carbon source, glucose, xylose, mock hydrolysate, or actual hydrolysate was added to fermentation media at the desired initial concentration based on experimental design. To ensure anaerobic fermentation, CO_2_ was sparged overnight before bacterial inoculation. All these fermentations were started at an initial OD_600_ of 0.1 from the inoculum in fermentors.

The experimental design consisted of four rounds of fermentation. Firstly, in order to determine the inhibition effect of sugar levels on succinate production, different initial concentrations of xylose and glucose (40, 60, 80, and 100 g/L) were separately evaluated. Secondly, taking into account the most adequate xylose concentration (80 g/L) to produce succinate, a mock DAP-H and DDAP-H were designed, including different sugars at the correct ratios (based on Table 1) such as glucose, xylose, galactose, and arabinose and also compounds such as furfural, HMF, and acetates (the later at two different concentrations to mimic DAP-H and DDAP-H). In the last round of fermentation, the actual DAP-H and DDAP-H were utilized, diluting them down to a final sugar concentration of 80 g/L. The percentage of hydrolysate used to achieve those sugar levels was 56 % v/v.

### Fermentation conditions and sampling

All fermentations were carried out in 0.5-L working volume BioStat-Q Plus fermentors with 300 mL of growth media in duplicate for each treatment excluding 40–100 g/L glucose and xylose and “mock sugars + AA.” The pH was maintained at 6.8 via supplementation of 2.5 M Na_2_CO_3_. The temperature was controlled at 37 °C and the agitation at 300 rpm. During the fermentation, CO_2_ was sparged at 0.03 vvm.

Samples (~1.5 mL) from the fermentations were taken in aseptic conditions at various time points in order to follow bacterial growth, sugar consumption, and the production or uptake of other acids (e.g., succinic, formic, acetic, and lactic acid), inhibitors (e.g., furfural, HMF, phenols), and other compounds (e.g., furfuryl alcohol).

### Analytical methods

ODs were measured at 600 nm in a Spectronic 601 spectrophotometer (Milton Roy, Ivyland, PA, USA). Samples were then centrifuged and filtered through a 0.2-μm syringe filter before placing them in high pressure liquid chromatography (HPLC) vials. Samples were analyzed for carbohydrates and organic acids (succinic, formic, acetic, and lactic acid) via HPLC using the Shodex SP0810 carbohydrate column and the Biorad Aminex HPX-87H organic acids column.

Furfural, HMF, furfuryl alcohol, and HMF-alcohol were separated and quantified on an Agilent 1100 series HPLC equipped with a diode array detector (DAD). The wavelengths monitored were 250 nm to detect furfural and 225 nm for furfuryl alcohol, HMF and HMFA. Samples and standards were analyzed using an Agilent Zorbax SB-C18 5 um 4.6 × 250 mm with a guard column. A mobile phase of acetate buffer (12.5 mM, pH = 4.5) and acetonitrile (4:1) was run isocratic at 1.0 mL/min for 10 min. Concentrations for the standards in acetate buffer were <1.0 mg/mL and standard curves were generated and used for quantitation. Samples were prepared by diluting 0.4 mL of culture supernatant with 0.4 mL of acetate buffer (25 mM, pH = 4.5) and 0.2 mL of acetonitrile. The flowrate was 1 mL/min, column compartment temperature of 25 °C, and injection volume was 10 µL.

### Analysis by HPLC diode array detector and electrospray ionization-tandem mass spectrometry

Individual chemical standards of 4-hydroxy benzaldehyde, homovanillic acid, vanillic acid, caffeic acid, syringic acid, syringaldehyde, coumaric acid, ferulic acid, and sinapic acid were purchased from Sigma-Aldrich, St. Louis, MO. HPLC solvents and modifiers consisted of deionized water (Barnstead Easy PureII, Waltham, MA), acetonitrile (Fisher HPLC grade), and formic acid (Sigma-Aldrich).

Analysis of samples was performed on an Agilent 1100 LC system equipped with a G1315B diode array detector and an Ion Trap SL (Agilent Technologies, Palo Alto, CA) mass spectrometer (MS) with in-line electrospray ionization (ESI). Each sample was injected undiluted at a volume of 50 μL into the LC/MS system. Primary degradation compounds were separated using reverse-phase chromatography on an YMC C30 Carotenoid 0.3 μm, 4.6 × 150 mm column (YMC America, Allentown, PA) at an oven temperature of 30 °C. The HPLC method was adapted from prior approaches [6, 56], with the solvent regime consisting of eluent A) water modified with 0.03 % formic acid, and eluent B) 9:1 acetonitrile and water also modified with 0.03 % formic acid. At a flow rate of 0.7 mL/min, the gradient chromatography was as follows: 0–3 min, 0 % B; 16 min, 7 % B; 21 min, 8.5 % B; 34 min, 10 % B; 46 min, 25 % B; 51–54 min, 30 % B; 61 min, 50 % B; and lastly 64–75 min, 100 % B before equilibrium.

Flow from the HPLC–DAD was directly routed to the ESI–MS ion trap. The DAD was used to monitor chromatography at 210 nm for a direct comparison to MS data. Source and ion trap conditions were calibrated with Agilent ESI-T tuning mix (P/N:G2431A), while tuning parameters were optimized under negative-ion mode by direct infusion of standards for major contributing compounds. MS and MS/MS tuned parameters are as follows: smart parameter setting with target mass set to 165 Da, compound stability 70 %, trap drive 50 %, capillary at 3500 V, fragmentation amplitude of 0.75 V with a 30–200 % ramped voltage implemented for 50 ms, and an isolation width of 2 m/z (He collision gas). The ESI nebulizer gas was set to 60 psi, with dry gas flow of 11 L/min held at 350 °C. MS scans and precursor isolation-fragmentation scans were performed across the range of 40–350 Da.

Quantitation of sample compounds was performed by the addition of 3,4-dihydroxybenzoic acid as an internal standard to adjust for analysis shifts. External analytical standards of the sample compounds were used to construct calibration curves for quantitation. All degradation compounds were quantified by LC/MS ion trap.

### Calculation of succinate yields, succinate productivity, and succinate maximum specific productivity

Succinic acid yields cannot be calculated by merely dividing the final SA titer by the initial sugar concentration. This is mainly due to extensive dilution caused by the constant addition of neutralizing liquid base (2.5 M Na_2_CO_3_) whereby the fermenter volume increases with time. In addition, compensation should also be made for the removal of substrate/products via sampling. Accordingly, the volume of base added was calculated using the dissociated protons from succinic, acetic, and formic acid formed. Compensation for sampling was made by using an average sample size of 1.5 mL. The fermenter volume was found to increase between 12 and 19 %. The yield values reported in Fig. 6 include these adjustments and report the actual mass of SA formed per mass of glucose consumed. Succinate titers (g/L) were not corrected by the dilution since those results are the actual data for downstream processes. Productivity (g/L-h) was calculated as succinate production (g/L) between the hours of fermentation at each time point. Maximum specific productivities (g/L-h) were calculated from those intervals of time where succinate productivity was maximum. Those time intervals are detailed in Fig. 6 legend.

## Additional file

10.1186/s13104-016-1842-8 Supporting information.

## Abbreviations

AA: acetic acid
DAP: dilute acid pretreatment
DAP-H: dilute acid pretreatment hydrolysate
DDAP-H: deacetylated, dilute acid pretreatment hydrolysate
HMF: hydroxymethyl furfural
HPLC: high pressure liquid chromatography
SA: succinic acid
