# Supplementary material for: Succinic acid production on xylose-enriched biorefinery streams by Actinobacillus succinogenes in batch fermentation
Source: Biotechnol Biofuels. 2016 Feb 2;9:28. doi: 10.1186/s13068-016-0425-1 (PMC4736274; doi:10.1186/s13068-016-0425-1)
Supplement: Supplementary file 1 — 10.1186/s13104-016-1842-8 Supporting information. [file 13068_2016_425_MOESM1_ESM.pdf]

Supplementary material

Succinic acid production on xylose-enriched biorefinery streams by *Actinobacillus succinogenes* in batch fermentation

Davinia Salvachua[1‡], Ali Mohagheghi[1‡], Holly Smith[1], Michael Bradfield[2], Willie Nicol[2], Brenna Black[1], Mary J. Biddy[1], Nancy Dowe[1,*] & Gregg T. Beckham[1,*]

1. National Bioenergy Center, National Renewable Energy Laboratory, Golden CO 80401
2. Department of Chemical Engineering, University of Pretoria, Pretoria, South Africa

[‡] DS and AM contributed equally to this work.
[*] Corresponding authors: nancy.dowe@nrel.gov; gregg.beckham@nrel.gov

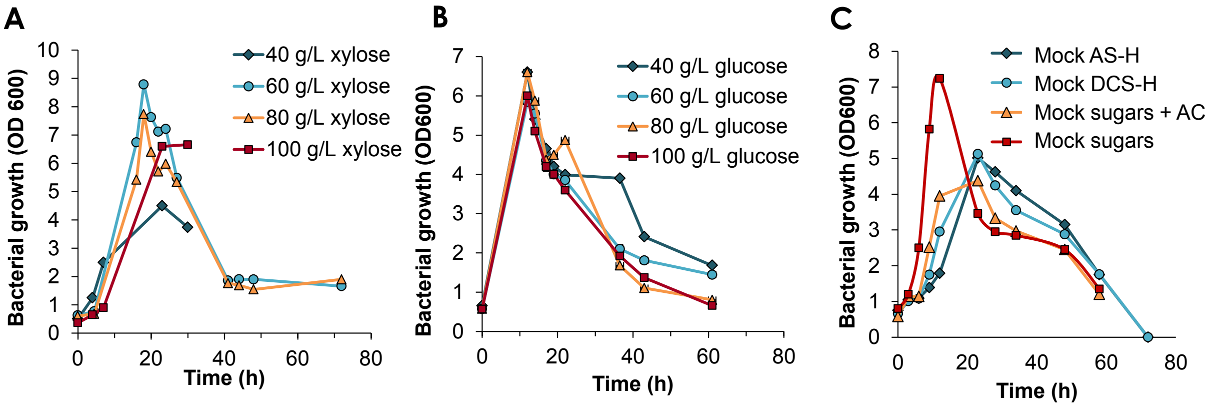

Figure S1. Bacterial growth (OD600) in (A) xylose, (B) glucose, and (C) mock media fermentations by *A. succinogenes.*

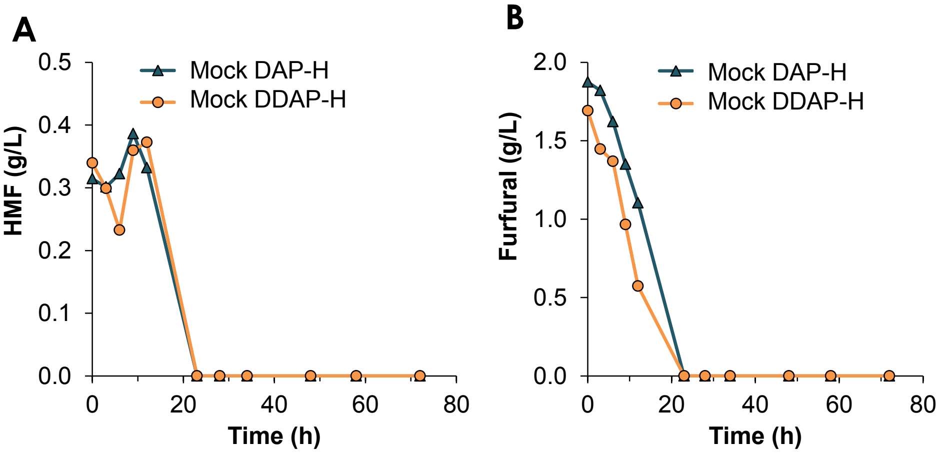

Figure S2. Conversion of (A) HMF and (B) furfural by *A. succinogenes* in mock DAP-H and DDAP-H.

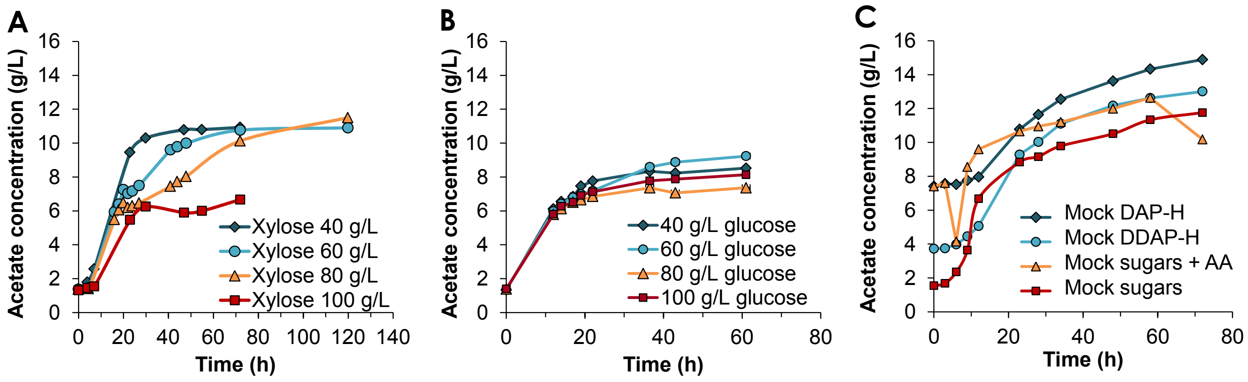

Figure S3. Acetate concentration in (A) xylose, (B) glucose, and (C) mock media fermentations by *A. succinogenes.*

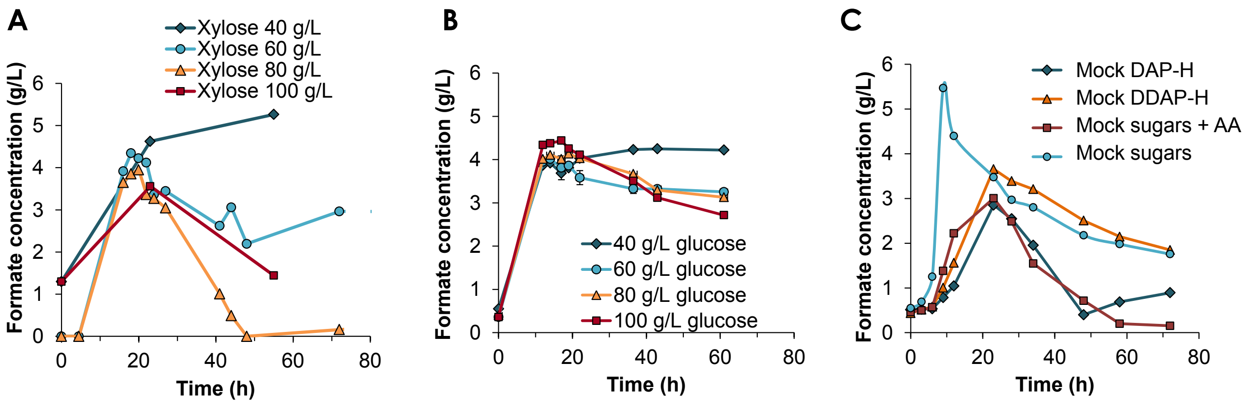

Figure S4. Formate metabolism in (A) xylose, (B) glucose, and (C) mock media fermentations by *A. succinogenes.*
